# Consulting the experts: young people's experiences of a school-based mental health literacy program

**DOI:** 10.1080/00049530.2025.2478089

**Published:** 2025-03-19

**Authors:** Alexandra Marinucci, Christine Grové, Kelly-Ann Allen, Bich Ngoc Hsu

**Affiliations:** a School of Educational Psychology and Counselling, Faculty of Education, Monash University, Clayton, Victoria, Australia; b School of Health and Biomedical Sciences, STEM College, RMIT, Melbourne, Victoria, Australia; c Fulbright Association, Canberra, New South Wales, Australia

**Keywords:** Youth, mental health literacy, school, perspectives, experience, program

## Abstract

**Objective:**

The prevalence of mental health problems among youth is high, and help seeking behaviour is low. Mental health literacy (MHL) has been suggested as a factor to enhance mental health knowledge and increase help seeking and coping behaviours. Schools have been recognised as an ideal environment for MHL programs to be delivered to reach a wide range of youth. This study aimed to understand the perspectives and experiences of secondary school students after participating in a MHL program, and how findings aligned with the theoretical model the program is based on.

**Method:**

Thirty-eight participants aged 12–16 years provided feedback through an open-ended questionnaire. The questionnaire comprised seven items to gather participants’ experiences and perspectives on the MHL program, including usefulness, ideas for program improvement, impact, and coping strategies. This study was preregistered on the Australian New Zealand Clinical Trials Registry, Registration Number: ACTRN12621000325808.

**Results:**

Reflexive thematic analysis generated three themes and three subthemes from the findings: 1) A safe environment, 2) Perceived positive impact, 2.1) Attitudes towards mental health, 2.2) Coping strategies, 2.3) Need for MHL, and 3) Suggestions for program improvement.

**Conclusions:**

These findings indicate that the school-based program meets the current MHL needs of young people, aligns with the MHL Child Focused theoretical model, and was viewed as beneficial to be incorporated into the education system in future.

## Key Points

**What is already known about this topic:**

1. Mental health literacy (MHL) is growing in research as a preventative approach to mental illness.
2. Program evaluation requires understanding participant perspectives, program alignment with theory, and experiences of the program.
3. Little research has been conducted exploring the experiences and views of young people following participation in MHL programs.

**What this topic adds:**

1. From the perspective of youth, their MHL improved following participation in a school-based MHL program.
2. Young people voiced positive views of learning practical mental health knowledge in a safe environment.
3. This study demonstrates how young people can be included in the evaluation of programs, and the importance of championing youth voice in research and supporting their needs.

Mental health difficulties are increasingly prevalent across all age groups, with young people particularly affected (Brennan et al., 2021). Alarmingly, suicide is the leading cause of death among 15- to 24-year-olds in Australia (Australian Institute of Health Welfare, 2022; Glenn et al., 2020). Additionally, many young people lack adequate knowledge about mental health issues (Gulliver et al., 2010; Radez et al., 2020). Research indicates that only about 50% of young people can correctly identify depression, and even fewer can recognise anxiety disorders (Coles et al., 2016). These trends highlight the urgent need to equip individuals, especially youth, with tools to develop and maintain good mental health through improved mental health literacy (MHL). While there has been growth in the development and evaluation of MHL programs (Marinucci, Grové, & Allen, 2022; Seedaket et al., 2020), a significant gap remains: these initiatives often fail to incorporate the perspectives of their primary beneficiaries – young people themselves. To address this gap, the present study employed a qualitative approach to examine an evidence-based MHL school program through the lens of youth participants. The Youth Education and Support (YES) program, adapted for Australian schools and informed by the MHL Child Focused model (Bale et al., 2020), served as the focus of this investigation. The study aimed to understand participants’ experiences, perceived benefits, and suggestions for improvement of the YES program. Furthermore, recognising that programs grounded in theory tend to have a stronger evidence-base (Durlak, 2003; Hage et al., 2007), this study also explored how well the YES program aligned with the MHL Child Focused model, as reported by participants.

## Mental health literacy

The concept of mental health literacy (MHL) has evolved significantly since its inception. Originally defined by Jorm et al. (1997) as knowledge primarily related to mental disorders, contemporary research has broadened the scope of MHL to encompass a more holistic understanding of mental health (Jorm, 2020; Kutcher et al., 2016). This expanded conceptualisation of MHL now includes help-seeking behaviours, recognition of mental health changes, awareness of supports, the development of adaptive coping strategies, resilience, and fostering positive attitudes towards mental disorders (Bale et al., 2020).

**Figure 1.**
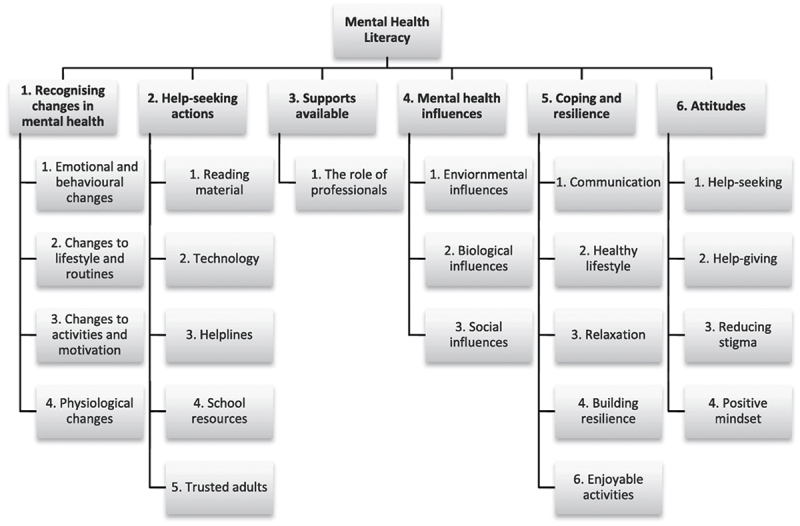
Mental health literacy child focused model (Bale et al., 2020).

Reprinted from Children and Youth Services Review, Vol 109, Justine Bale, Christine Grové & Shane Costello, Building a mental health literacy model and verbal scale for children: Results of a Delphi study 104667, Copyright (2020), with permission from Elsevier.

A significant contribution to MHL is the *MHL Child Focused model* (Figure 1; Bale et al., 2020), which emerged from a Delphi study synthesising expert opinions on factors crucial to young people’s MHL. The model outlines six main youth-specific components of MHL, including: recognising changes in mental health, help-seeking actions, supports available, mental health influences, coping and resilience and attitudes. Identifying constructs of MHL provides areas that programs could target to increase MHL. Despite its potential, there has been limited research on how theoretical models like this align with real-world MHL programs and youth experiences (Davis et al., 2015). This gap is particularly noticeable in school-based settings, where the practical application of such models remains understudied. This raises a growing need to understand how theoretical MHL frameworks translate into effective interventions that resonate with young people’s experiences and needs. This understanding is important for developing and refining evidence-based MHL programs that can effectively improve youth MHL (Francis-Oliviero et al., 2023; O’Mara & Lind, 2013). Exploring the alignment between theoretical models and program outcomes from the perspective of young participants themselves could provide valuable insights into the relevance of current MHL approaches in school settings.

## Mental health literacy programs

Schools appear to be an appropriate and suitable context for the provision of MHL to a large population of young people (Conley & Durlak, 2017). Despite requests from young people for more education on mental health in schools, there seems to be an insufficient amount of relevant programs in place (Marinucci, Grové, & Rozendorn, 2022). Youth express a desire for programs to cover topics focusing on how to identify and manage mental ill health symptoms, gain practical coping strategies, understand support options and maintain good mental health (Chandra & Minkovitz, 2007; Marinucci, Grové, & Rozendorn, 2022; Tharaldsen et al., 2017; Woolfson et al., 2009).

Initiatives have been established to attempt to address the lack of MHL among young people (e.g., BeYou; Beyond Blue, 2021), yet mental health prevention school-based programs are limited (Dix et al., 2020; Marinucci, Grové, & Allen, 2022). Reviews of school-based MHL programs have found many studies have low inclusion of stakeholder involvement (Amado-Rodríguez et al., 2022; Seedaket et al., 2020), with little research conducted in an Australian school setting (Marinucci, Grové, & Allen, 2022). This further implicates the evidence-base and uptake of school-based MHL programs (Mihalic & Elliott, 2015).

## Implementation science in program evaluation

Evidence-base, sustainability and generalisability of programs are driven by implementation science, “the scientific study of methods to promote the systematic uptake of research findings and other evidence-based practices into routine practice, and, hence, to improve the quality and effectiveness of health services” (Eccles & Mittman, 2006, p. 1). From development to evaluation, implementation science is impacted by the theoretical framework on which programs are based on (Durlak & DuPre, 2008; Nilsen, 2015); thus, alignment with theory is crucial. Though programs are commonly aligned with a theory during development (Moir, 2018), little research has assessed program-theory alignment at the evaluation stage. Investigating whether experiences of programs map onto the intended theoretical framework provides further support that the program is evidence-based. Implementation science is also affected by participant receptiveness and engagement.

Participant receptiveness and engagement are a major determinant of positive outcomes from school-based mental health programs (Rojas-Andrade & Bahamondes, 2019), and measuring this allows for participant perspectives and experiences to be considered (Hill & Erickson, 2019). Whilst quantitative measures can be useful to address program effectiveness, this does not allow for an in depth evaluation of individual experiences and perspectives, and insight for future development of programs based on current needs (Shiner et al., 2008). Youth engagement is enhanced when they are interested in the information they receive (Flowerday & Shell, 2015), and it has been argued that youth receptiveness is the strongest predictor of change from school-based mental health programs (Macklem, 2014; Rojas-Andrade & Bahamondes, 2019). A possible method to assess whether a program satisfies young people’s needs and receptiveness is to directly seek their feedback via qualitative surveys (Teng et al., 2017).

## Youth participation and perspectives

Typically, key stakeholders (e.g., teachers, parents) are consulted during the development and implementation of programs, however the perspectives of young people are often not emphasised (Forshaw & Woods, 2022; Grové et al., 2020). Though school-based programs directly involve young people, they are rarely asked about their experiences and needs during the implementation process of these programs (Bee et al., 2014). Young people have a right to contribute to making decisions that directly impact them and have demonstrated that they are able to effectively identify and communicate their needs (Grové et al., 2020; United Nations, 1989). Mitra and Gross (2009) highlighted that youth have unique experiences and opinions in schools that are distinct from those of adults, thus collaboration can enrich the program content. Furthermore, when youth are allowed to be involved in the development and evaluation of programs, they are more likely to feel heard, respected, and supported, and more likely to engage in the behaviours promoted by the programs (Jolivette et al., 2015). A key method to understand youth experiences and perspectives of programs is through exploring participant receptiveness and engagement (Carroll et al., 2007). This is critical to the acceptability and dissemination of programs in the future (Feely et al., 2018).

Studies of mental health and MHL programs have investigated youth engagement and perspectives of programs following participation, for example, the Help Out a Mate program (Wynters et al., 2021), and the OurFutures: Mental Health program (Peters et al., 2023). Gaining perspectives of young people provided valuable insight into program experience, for example, how likely they would use the skills learnt in future, and how relevant the program was for their lives. However, the Help Out a Mate program took place within sports clubs, and the OurFutures: Mental Health is an online only program. To the researcher’s knowledge, limited research exists exploring the perspectives and experiences of young people participating in a school-based MHL program.

## The current study

Young people’s opinions have not been elicited to discuss school-based MHL programs and understand their experience. The current landscape of school-based MHL programs lacks exploration of participant experiences and perspectives, and alignment with theory after implementation (Amado-Rodríguez et al., 2022; Dix et al., 2020; Marinucci, Grové, & Allen, 2022). Thus, there is a need to enhance understanding of program experience from the perspectives of participants. Given that the YES program was adapted for an Australian context and that this is the first time it has been implemented and evaluated, the ability to explore how the program aligns with both established theory and the needs of young people was critical for future development and growth of the program.

The present study aimed to understand the receptiveness, perspectives and experiences of youth participating in the YES program, guided by the research question:

What are the perspectives and experiences of youth of the YES program in addressing their MHL needs?

## Method

### Design

The current study used a qualitative methods design. The quantitative findings of the effects of the MHL program are discussed elsewhere (Marinucci et al., 2024). For the purpose of the current study, adherence and program implementation are not explored, however is addressed in Marinucci et al. (2024). Participants who had completed the MHL program completed a seven-item open-ended questionnaire to share their experiences and perspectives of the program. Data were analysed using reflexive thematic analysis.

### Participants

Eighty-four secondary school students participated in the MHL program from February to August 2022 (Marinucci et al., 2021, 2024), and 38 participants completed the evaluation measure at the end of the program and were included in this study. The inclusion criteria were 12–16-year-old students (Grades 7 to 10) who were attending a mainstream secondary school and were competent in written and spoken English.

Although 84 participants completed the YES program, pre-post measure attrition and incomplete data (as outlined in Marinucci et al. (2024) with challenges to recruitment and retention of participants) resulted in an overall smaller sample size for analysis. Attrition from the program was affected by students missing sessions due to illness, particularly as during the time of implementation cases of COVID-19 and close contacts (e.g., household members) were required to isolate for 7 days. Additionally, due to the nature of the busy school environment, other commitments or academic classes requiring attendance impacted the attrition from the program. Table 1 details the demographics of the sample. Participating schools were located in the 5th quintile, 4th quintile, and 3rd quintile on the Index of Relative Socio-economic Advantage and Disadvantage (1st quintile = most disadvantaged, 5th quintile = most advantaged; Australian Bureau of Statistics, 2018).

**Table 1.** Demographics of participants (*N* = 38).

Demographic Characteristics	*n*	Proportion (%)
Age (yrs)		
Mean	14.1	–
SD	1.0	–
Range	12–16	–
Gender		
Male	12	32
Female	26	68
Grade		
7	5	13
8	3	8
9	30	79
Ethnicity		
Asian	1	3
Asian-Australian	1	3
Australian	26	68
Italian	2	5
Italian-Australian	2	5
Italian-Greek	1	3
Macedonian-Turkish	1	3
Not provided	4	11
School		
Government	8	21
Non-government	16	42
Catholic	14	37

### The YES program

The Youth Education and Support (YES) program was first established by Joanne Riebschleger based on over 12 years of research (Riebschleger, 2013; Riebschleger et al., 2009, 2017, 2019), and has been adapted for an Australian context based on the MHL Child Focused model proposed by Bale et al. (2020), and aligned with the Australian Health and Physical Education curriculum and principles from the Universal Design for Learning (Marinucci et al., 2021). The YES program focuses on stress, coping strategies, mental illness and recovery, depression and anxiety, resilience, help seeking and support, stigma, and mental illness in families. Whereas the original version of the program focused on substance misuse, as this area is already addressed through the Australian health education curriculum (Australian Curriculum, Assessment and Reporting Authority, 2018), the Australian YES program instead focused on help-seeking actions, coping and resilience according to the MHL Child Focused model (Bale et al., 2020). It is comprised of 10 sessions, each lasting approximately 50 min, with a detailed discussion of how the program was adapted outlined in Grové et al. (2023). Recent quantitative evaluation of the YES program demonstrated that participants’ MHL significantly increased following the program, and participants were more likely to help seek for suicidal ideation, providing promising initial evidence for youth MHL intervention in the school setting (Marinucci et al., 2024).

Evaluation and analysis of the YES program was informed by the field of implementation science (Eccles & Mittman, 2006). Methods of youth engagement and participation were informed by the P7 model with the hope that due to their active engagement, young people might find the program relevant and practical to apply the obtained knowledge into their everyday life (McCabe et al., 2023). According to the P7 model, youth participation can be enacted through considering different domains that affect young people’s involvement in program design, implementation, and evaluation (Cahill & Dadvand, 2018).

The P7 model was chosen to guide youth participation in this study as it holistically considers including young people through aspects of research as a process rather than a one-off event, gives young people a choice to participate, and participation is driven by their capabilities (Duke et al., 2022). Youth participation was enacted in the current study according to the P7 Model. The domains and how the P7 model was applied to youth participation in the current study is detailed in Table 2.

**Table 2.** Application of the P7 model to the current study.

Domain	Definition	Application to current study
Purpose	Articulating how and why young people are involved, and providing them with opportunities to play an active role	Explicitly seeking feedback from the youth participants and highlighting their experience would play an important role in further refinement of the program
Positioning	The way that young people are framed for what is possible in their contribution	Young people positioned as recipients and co-contributors to the program
Perspectives	The capacity in which young people’s voices are included	No exclusion criteria for participating
Power	Identifying how power relations are managed between young people and adults	Young people creating values and expectations for the group and clearly explaining that participation is voluntary
Protection	The practices included to promote and enhance safety	Ensuring confidentiality and the limits to confidentiality
Place	Acknowledging that place or context affect participation	The program taking place in a setting accessible to young people
Process	The structure that enables participation and inclusion	Providing anonymity of questionnaires to foster unbiased feedback

Youth participation in research was facilitated from development to evaluation of the MHL program, through a needs analysis (Marinucci, Grové, & Rozendorn, 2022) prior to developing and adapting YES to an Australian school setting, and through the current study.

### Materials and procedure

Ethical approval was obtained from the relevant University Human Ethics Research Committee and governing school organisations. The recruitment process involved researchers emailing invitation letters to school principals, posting advertisements and media releases through social media platforms. Recruitment began in 2021 with 19 schools expressing interest in participating, however due to COVID-19 implications, implementation of the program was re-scheduled to 2022 with four schools having capacity to take part in the program. Written informed consent forms were obtained from participants, parents, and school principals.

The program evaluation form was distributed in the form of a voluntary, anonymous online questionnaire through the Qualtrics (2022) platform. Participants used their personal devices to fill out the forms, and those without a device were provided one by the school or the facilitators. The online questionnaire was administered in the final session of the program and comprised seven open-ended questions. The questionnaire was developed by the researchers based on previous literature (Marinucci, Grové, & Allen, 2022; Peters et al., 2023; Wynters et al., 2021) and the evaluation questionnaire used in the original YES program (Riebschleger et al., 2019). The questionnaire aimed to gather participants’ experiences and perspectives on the MHL program, including what was useful, their ideas for program improvement, the program’s impact, and their preferred coping strategies (see Table 3). All responses were anonymous and pseudonyms were used when coding participants’ data during thematic analysis.

**Table 3.** Evaluation questionnaire.

1. What were some of the things we did in the YES program? 2. What did you like about the YES program? 3. What would make the YES program better? 4. When you think about the whole program, what did you learn most in the YES program? 5. To what extent has the YES program made a difference in your life? Examples? 6. What kind of coping strategies do you think work best for most youth like you? 7. What would you tell other youth about YES?

### Data analysis

Qualitative data were analysed using reflexive thematic analysis. Thematic analysis is a method used to identify, organise, and understand themes within a dataset (Braun & Clarke, 2006, 2021). This involves six steps (Braun & Clarke, 2021):

1. Familiarisation with the data set: Reading the qualitative transcript several times.
2. Coding: Exploring patterns in the data and developing codes and a coding frame.
3. Generating initial themes: Clustering connected codes into themes.
4. Developing and reviewing themes: Ensuring the theme conveys importance.
5. Refining, defining, and naming themes: Outlining the scope and concept of the theme.
6. Writing: Providing a concise account of the themes.

This study adopted a deductive approach to generate themes from participants’ data. Reviewers of the data were experienced in educational and developmental psychology in both clinical and research capacities, with extensive experience in qualitative research and thematic analysis. For the analysis, one reviewer first transferred the data from the questionnaires into a spreadsheet and shared this with a second reviewer. Two reviewers independently coded the data to capture the broad semantic and conceptual meaning of the data. Independent coding helped to reduce the risk of bias and strengthen the reliability of the findings (Braun & Clarke, 2021). A coding frame was generated by the two reviewers to add systematic engagement and analytical insight to the meaning of the data across the entire dataset (Braun & Clarke, 2021), and a third independent coder coded the data to ensure the codes reliably captured the essence of the data. Disagreement was managed through discussion between the two reviewers and inter-coder reliability between the two reviewers was analysed using Krippendorff’s Alpha (α = .93), indicating good reliability (Hayes & Krippendorff, 2007) and little disagreement on coding of the data. Codes of similar patterns were categorised into themes, and the two reviewers collaborated and collated their thematic analysis to come to finalise the themes. A third reviewer evaluated the final themes generated to ensure the themes conveyed importance and the complex concepts underpinned in the qualitative data. Finally, the themes were refined to generate three themes and three subthemes from the findings.

Though all seven specific questions were responded to in the questionnaire, each did not necessarily map onto one particular theme. Instead, there was overlap in responses to questions during coding and analysis, with some questions mapping onto one specific theme. For example, for the question “What kind of coping strategies do you think work best for most youth like you?” mostly mapped onto subtheme 2.2 of coping strategies.

## Findings

### Thematic analysis

**Figure 2.**
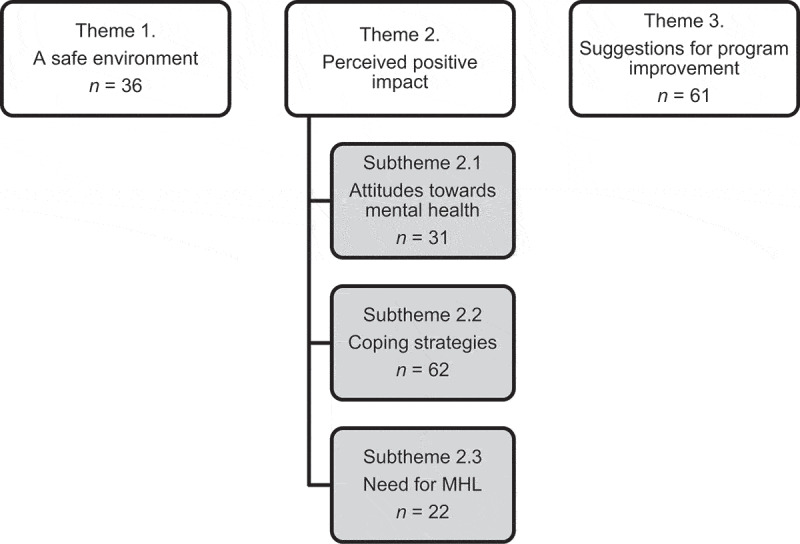
Themes and subthemes generated.

Through reflexive thematic analysis of the data, three themes emerged: 1) A safe environment, 2) Perceived positive impact (with three subthemes), and 3) Suggestions for program improvement. Figure 2 presents the themes, subthemes and number of quotes per theme/subtheme.

#### Theme one: a safe environment

The first theme centred around how the MHL program provided a safe environment to learn about mental health. A sense of belonging was reported by participants. For example, students noted “How you can say anything without judgement. The environment in the room is always upbeat” and “I enjoyed getting to know the group … feels like a safe space. I liked learning and being able to ask questions”. Collaborating with peers with open communication in a fun environment was also mentioned by the participants.

#### Theme two: perceived positive impact

The second theme explored the impact of the MHL program on the participants and perception of need for MHL based on their self-report. Three subthemes emerged: attitudes towards mental health, coping strategies and need for MHL.

##### Subtheme 2.1: attitudes towards mental health

Attitudes towards mental health were most often reported as changed as a result of the MHL program: “the YES program has taught me how other people with mental illness are feeling and what not to say, but also what to say”. These attitudes also filtered to how young people perceived seeking help: “asking for help is ok” and “who we can contact and how to deal with stress, anxiety, depression”.

Awareness of the difficulty for some experiencing mental illness was identified: “Telling someone to just get help isn’t actually that easy for some people”. Participants reported they now understood the difference between depression and anxiety, and one participant reported they recognised they may be suffering from depression. Knowledge that participants were not alone in their mental health challenges and the ability to talk about mental health with others were identified as significant changes: “I feel less ashamed about my mental health struggles”, “It has helped me gain perspectives, I know strategies to cope … and that I am not alone”, and “It has made a huge difference. I used to keep things to myself a lot but now I talk to my family and friends more”.

##### Subtheme 2.2: coping strategies

Increased knowledge of how to cope with stress through different strategies, understanding mental health holistically, and having a different mindset were noted as positive outcomes of the program by most participants. Through teaching engaging and relevant content, participants perceived their MHL to have increased: “How much I have learnt about a mental illness. The coping strategies … learning where I can get help” and “Learning new ways of coping and that there are others out there that are in a similar situation and are there to help”. Additionally, participants reported that their use of positive coping strategies had increased and felt that the following coping strategies work best for young people: exercising and sport, listening to music, using fidget toys or putty, taking time away from others, getting fresh air, focusing on positive things, breathing exercises and mindfulness activities, talking with friends and family, journaling, reading, and eating.

##### Subtheme 2.3: need for MHL

Finally, the MHL program had an impact on how young people perceived the need for MHL: “It was helpful for me and I think everyone should get educated on this topic” and “Its [sic] really worth participating in and beneficial to anyone”. Participants reported that they would tell other young people that the MHL program was fun and helpful and that they can learn about mental health to help themselves or others: “it is good to go to so you can learn about things that may help you now or maybe later” and “You will learn so much and hand that over to other people”.

#### Theme three: suggestions for program improvement

The final theme generated the content that was most important and remembered by participants at the end of the program, and what they perceived would improve the MHL program in the future. Participants recalled that during the program they did activities focused on managing mental health symptoms, understanding mental health and mental illness, improving coping skills, learning how to seek help and help others, improving attitudes towards mental health, and identifying their values. It was reported that discussions and reflections were incorporated into the sessions, with multimodal activities included. However, they reported that the program could be improved by increasing the number of interactive activities, providing fidget toys to use during the session, and having more incentives (such as lollies). Logistical set-up of the group was also mentioned, including having small groups, longer or more sessions, more active group discussions, the ability to have private conversations with facilitators, and to sit in a circle. Providing more explanations of different mental illnesses was also suggested, as well as a summary of all sessions. These reports indicate participants demonstrated a good level of engagement and relevance to the program, with improvements to the delivery of content highlighted.

## Discussion

This study aimed to understand youth perspectives and experiences of the YES program to address the current lack of youth voice in program evaluation research. This was guided by the research question: What are the perspectives and experiences of secondary students on the YES program in addressing their MHL needs?

Young people showed engagement and appreciation for the mental health information taught in the program within the accessible and safe school environment, and many agreed that they benefited from the program. Additionally, young people’s feedback provided further evidence for the MHL program aligning with the theoretical model it was developed on, the MHL Child Focused model (Bale et al., 2020). Given the current shortage of evidence-based school-based programs, these results suggest that the MHL program is a promising MHL program to deliver to secondary schools (Dix et al., 2020; Marinucci, Grové, & Allen, 2022).

### Youth perspectives: a safe environment

Overall, youth enjoyed the environment of the program and viewed it as interesting, safe, and informative. This allowed them to freely participate in different activities, express themselves, and share their ideas, thus fostering a sense of belonging. As suggested by the principles of the Universal Design for Learning, these are beneficial features that promote youth engagement, and hence can increase the likelihood of learning and making use of the learned materials (Levey, 2021). For some youth, this program was the first time they were exposed to in-depth learning of different mental health concepts. Therefore, the information taught helped them to develop a comprehensive understanding of mental health and what was needed to maintain their personal mental health.

The program provided useful educational information and enhanced a sense of belonging among young people. Social factors played an important role in helping young people feel more comfortable in sharing their opinions and problems, thereby increasing their engagement in the learning materials (Pechmann et al., 2020). Social inclusion and a sense of belonging are relevant to MHL programs, as young people are more likely to experience positive school outcomes when they feel they belong (Arslan et al., 2021; Berger et al., 2022). Belonging can also be promoted by schools understanding student needs holistically, providing opportunities for students to connect with peers in a safe environment and increasing students’ ability to manage and cope with adversity (K. Allen et al., 2017; K.-A. Allen et al., 2018). Young people in the YES program experienced a sense of belonging, reflected in their appreciation of the non-judgemental environment and their ability to openly discuss their thoughts and feelings. This allowed them to foster relationships, increase their engagement, and collaborate with others in an atmosphere they perceived as safe and accepting. This kind of belonging encourages individuals to actively participate, seek help, and express their concerns or questions freely, which are crucial elements in the process of learning and understanding mental health concepts.

### Youth perspectives: positive impact

Previous research identifying areas recommended by youth to be taught in schools were covered in the MHL program, including mental illness signs and symptoms, coping strategies, stigma, prevention, resilience, and help seeking (Chandra & Minkovitz, 2007; Marinucci, Grové, & Rozendorn, 2022; Tharaldsen et al., 2017; Woolfson et al., 2009). After completion of the MHL program, youth reported increased knowledge of mental health and practical coping and help seeking strategies. Young people recognised the impacts of stigma, were aware of ways to prevent it and learned the appropriate methods to support others. These are seen as major components of the Bale et al. (2020) MHL Child Focused model, suggesting that the program successfully aligned with the theoretical model.

Youth described the program positively, and there was recognition amongst young people of the need for mental health education. The impact of the program was seen through reported improvements in coping, help seeking, and attitudes towards mental health. Youth appeared to apply appropriate coping strategies in real life and referred to their use of the “coping toolbox” created in the program. Most young people believed that emotion-focused strategies (e.g., engaging in activities they enjoyed) were helpful. Others preferred to have someone to share their struggles with, such as family members or friends. Young people appeared to have developed their own adaptive coping strategies aligned with open communication, healthy habits, relaxation, and engagement in preferred activities. The variety of coping strategies young people learned from the MHL program to obtain and maintain good mental health supports how mental health can be promoted without a sole focus on illness.

Youth reported that they became more comfortable in seeking help for themselves and learned ways to appropriately help others. This readiness to seek help may be attributed to an increase in understanding of mental illness signs and symptoms and improved articulation of their feelings (Aguirre Velasco et al., 2020; Radez et al., 2021). Family and friends were the main sources that participants noted they would seek support from, aligning with research demonstrating that youth tend to rely on informal sources of support (Tiller et al., 2021). Although health professionals such as psychologists and school counsellors were not explicitly mentioned, some young people stated they were aware of the professionals whom they could contact for help. While parents and friends are crucial for social and emotional support, professional assistance may be necessary in approaching more severe mental illnesses. It is highlighted in Bale et al. (2020) that youth should understand the roles of mental health professionals and when to seek this support.

Increased positive attitudes towards mental health and awareness of mental health stigma were reported by participants. Stigma is a major risk factor discouraging one from disclosing one’s experiences and asking for support (Aguirre Velasco et al., 2020; Tharaldsen et al., 2017). Therefore, the improved attitudes may help young people to feel more open to seeking help for themselves and giving help to others. Overall, the MHL program resulted in some positive changes, including a gain in mental health knowledge; increased confidence in asking for help and where to seek help when needed; ability to use their own coping strategies; and improved attitudes towards mental health.

### Youth perspectives: suggestions for program improvement

Young people offered multiple recommendations to improve the MHL program. A need for more sessions to expand on mental health information was requested, demonstrating that they valued the materials learned in the program and were eager to learn more about mental health. Although the current study was limited in time to fit within the school schedule, this may be achievable through incorporating the program into the school curriculum. Such a program was also recommended by young people in another study (Marinucci, Grové, & Rozendorn, 2022); however, further research is required to support this implementation suggestion. Other recommendations were given in relation to how the program is structured. For example, several young people expressed their desire for the group to sit in a circle instead of the usual classroom format to help them feel more comfortable and open to discuss, share, and connect with their peers. This is consistent with research identifying that seating arrangement in schools can impact student wellbeing and mental health (Bluteau et al., 2022) and is therefore a logistical solution that could foster more engagement in the program in the future. Altogether, this feedback is important in the refinement and retention of content of the MHL program, moving forward to ensure the needs of young people are appropriately adapted to.

### Alignment with theory

Mental health programs should be conceptualised according to framework or theory to support development of literature and strengthen the evidence-base (Durlak, 2003; Hage et al., 2007). Particularly for the field of health and mental health, research guides practice and thus is seminal in decision-making and tailoring programs to population needs (Kretlow & Blatz, 2011; Prasun, 2013). The MHL program was developed according to the MHL Child Focused model proposed by Bale et al. (2020), and findings and emerged themes from the current study indicate that the program has successfully aligned with this theory, as detailed in Table 4.

**Table 4.** Alignment of the MHL program with the MHL Child Focused Model.

Component	Findings
Recognising changes in mental health	Improved understanding of mental health, including recognising emotional and behavioural changes that could indicate change in mental health.
Help-seeking actions	Increased self-reported willingness to seek help from others. Increased confidence in helping others who may struggle with mental health difficulties. Engagement in materials related to help seeking knowledge such as websites, helplines, and school resources.
Supports available	Increased understanding of professional support available, as well as support within the school environment and how to seek this help (e.g., school psychologist referral pathway).
Mental health influences	Increased awareness of environmental, biological, and social influences of mental health.
Coping and resilience	Increased use of positive and healthy coping strategies, including enjoyable activities, relaxation, and lifestyle habits.
Attitudes	Positive attitudes towards mental health, and viewing mental health holistically (i.e., not solely the presence of mental illness). Increased empathy and understanding towards those who do experience mental illness.

Although this is based on self-reported qualitative data, the findings highlight that from the perspectives of young people, their ability to recognise changes in their mental health, use appropriate help seeking actions and understand the support available, acknowledge influences of mental health, incorporate positive coping strategies and build their own resilience to adversity, and have a positive attitude towards mental health have been improved by their participation in the MHL program. These are all components of the MHL Child Focused model (Bale et al., 2020) and derived from literature and expert views of core components of child and adolescent MHL. Mapping the perspectives and experiences of the participants onto the constructs of MHL according to theory and informed by implementation science (Durlak & DuPre, 2008; Nilsen, 2015) enabled the researchers to identify that program objectives are aligned with a theoretical foundation. Thus, the findings from the current study strengthen the evidence-base for the YES program.

### Study strengths, limitations, and future directions

A major strength is that this is the first study to seek to understand and include youth perspectives in the MHL program, and use their feedback to confirm that the program aligns to a theoretical model. Young people have not been given the opportunity to contribute their opinions to programs that have direct impact on them (Grové et al., 2020; Langhout & Thomas, 2010). Respecting the need for youth autonomy, the current study has attempted to involve their voices in both the development and implementation of the MHL program (Marinucci, Grové, & Rozendorn, 2022). This study also indicated that young people are competent in identifying their needs and engaging in research (Grové et al., 2020; Mannay et al., 2017). Additionally, the reliability of the interpretation of the findings is strengthened by the use of multiple coders for the qualitative data (Hayes & Krippendorff, 2007). A summary capturing the advice gained from the youth in this study for future recommendations is presented in Table 5.

**Table 5.** Summary of advice gained from youth.

Area	Advice
A safe environment	Presenting information in a range of formats and in an interesting way Providing a holistic understanding of mental health Creating an environment where youth can create meaningful relationships, connect, and work with peers Fostering a non-judgemental and welcoming space
Positive impact	Teaching youth about what mental health is, how mental illness impacts themselves and others, and how to cope with a mental health problem Providing practical coping and help seeking strategies with meaningful and engaging content Increasing positive attitudes towards mental health
Suggestions for program improvement	Delivering content over more than 10 sessions Sitting in a circle

There are a few limitations to this study. As data was collected in the form of a questionnaire, elaboration and clarification on responses was not possible. Future research may explore the use of focus groups to obtain a more comprehensive understanding of youth experiences in the MHL program. Additionally, while some suggestions were given by youth to improve the program, overall, these were few in number. Lack of critical feedback and overly positive responses may bias the results and are unhelpful for program refinement. To overcome this, complementing open-ended response questions with Likert scale questions may be necessary in future studies to determine whether needs are sufficiently met. Schools that agreed to participate were from overall higher socio-economic areas, thus results could be biased by higher household income, family educational level, occupation, and overall socio-economic advantage. Further research could investigate the impact of a MHL program for lower socio-economic areas to determine whether differences arise in these populations. Certainly, programs must be adapted to suit the needs of populations, therefore implementing a MHL program may provide further recommendations to ensure cultural and social sensitivity. Finally, the opt-in nature of the program may have had an impact on the results. Participants who opted in may have had an interest in mental health and therefore viewed the program as beneficial. Within a school environment, if the program were delivered to all students, including those who may not view mental health as important, participation and perspectives may be different. Future research could evaluate the receptiveness and acceptance of the MHL program at a larger scale, such as an entire year level, to gain more diverse perspectives.

## Conclusions

The inclusion of youth voice in the current study and investigation of young people’s perspectives and experiences of the YES program indicates improvement in youth MHL following participation in the program. As evidence that the program impacted participant’s daily life, young people noted finding the program helpful, informative, and engaging. The ability to learn conceptual and practical mental health knowledge in an open and positive environment was highly valued. Findings from the current study also highlight how the YES program aligned with the theoretical model on which it is based upon, the MHL Child Focused model (Bale et al., 2020). This further adds to the evidence-base supporting the YES program for targeting and improving youth MHL.

Given youth provided useful feedback and were willing to provide this suggests they are interested in the participatory nature of youth-centred research. Involving young people in research that directly affects them can contribute to increased engagement and acceptability of programs. This should be continued as the needs of youth change as they grow and the ecological system within which young people are positioned is constantly evolving. This study demonstrated how youth voice can be included in the implementation of school-based MHL programs, and supports young people in championing their MHL needs in a research and school context.

## Data Availability

The data that support the findings of this study are available from the corresponding author, AM, upon reasonable request.
